# The “Obiettivo Antibiotico” Campaign on Prudent Use of Antibiotics in Sicily, Italy: The Pilot Phase

**DOI:** 10.3390/ijerph17093077

**Published:** 2020-04-28

**Authors:** Martina Barchitta, Annalisa Quattrocchi, Andrea Maugeri, Maria Clara La Rosa, Claudia La Mastra, Guido Basile, Giovanni Giuffrida, Francesco Mazzeo Rinaldi, Giuseppe Murolo, Antonella Agodi

**Affiliations:** 1 Department of Medical and Surgical Sciences and Advanced Technologies “GF Ingrassia”, University of Catania, 95123 Catania, Italy; martina.barchitta@unict.it (M.B.); andrea.maugeri@unict.it (A.M.); mariclalarosa@gmail.com (M.C.L.R.); claudia.lamastra@unict.it (C.L.M.); 2 LaPoSS—Laboratory of Policies and Social Services, University of Catania, 95121 Catania, Italy; giovanni.giuffrida@gmail.com (G.G.); fmazzeo@unict.it (F.M.R.); 3 Department of Primary Care and Population Health, University of Nicosia Medical School, Ilia Papakyriakou, 2414 Engomi, Nicosia, Cyprus; quattrocchi.a@unic.ac.cy; 4 Department of General Surgery and Medical-Surgical Specialties, University of Catania, 95123 Catania, Italy; gbasile@unict.it; 5 Department of Social and Political Sciences, Bench S.r.l University of Catania, 95131 Catania, Italy; 6 KTH, Royal Institute of Technology, School of Architecture and the Built Environment, 100 44 Stockholm, Sweden; 7 Department of Health of the Sicilian Region, DASOE, 90145 Palermo, Italy; giuseppe.murolo@regione.sicilia.it

**Keywords:** prudent use antibiotics, campaign, communication, antimicrobial resistance

## Abstract

The issue of antimicrobial resistance (AMR) is a focus of the World Health Organization, which proposes educational interventions targeting the public and healthcare professionals. Here, we present the first attempt at a regionwide multicomponent campaign in Sicily (Italy), called “Obiettivo Antibiotico”, which aims to raise the awareness of prudent use of antibiotics in the public and in healthcare professionals. The campaign was designed by an interdisciplinary academic team, and an interactive website was populated with different materials, including key messages, letters, slogans, posters, factsheets, leaflets, and videos. The campaign was launched in November 2018 and, as of 21 December 2018, the website had a total of 1159 unique visitors, of which 190 became champions by pledging to take simple actions to support the fight against AMR. Data from social media showed that the audience was between 18 and 54 years of age, with a high proportion of female participants (64%). Interestingly, the LinkedIn page received more than 1200 followers, and Facebook 685 followers. The number of actions taken (pledges) by the audience was 458, evenly divided between experts (53%) and the general public (47%). Additional efforts are needed to reach more people, thus future efforts should focus on further promotion within the Sicilian region to sustain the engagement with the campaign.

## 1. Introduction

Antimicrobial resistance (AMR) is the ability of microorganisms to become increasingly resistant to an antimicrobial to which they were previously susceptible [1]. The process of AMR is a natural phenomenon but the inappropriate use of antibiotics accelerates the emergence and spread of resistance [1]. AMR is a serious threat to public health and infections caused by antibiotic-resistant pathogens and is associated with increased length of hospital stay, and results in substantial economic burden because of higher treatment costs and reduced productivity caused by sickness. Specifically, the problem is of high concern particularly in Italy where multidrug-resistant pathogens have been shown to be involved in severe healthcare-associated infections (HAIs) and frequently involved in outbreaks, especially in intensive care units [2,3,4,5,6,7]. The appropriate and responsible use of antibiotics could help to stop the phenomenon [2,3,4,5]. Information, attitudes, and action of the public are of vital importance in safeguarding the prudent use of antimicrobials [1]. Indeed, due to the widespread persistence of misconceptions about the nature and effectiveness of antimicrobials, education, training, and communication are an important part of the strategy to tackle AMR [1]. The problem of AMR is a focus of the World Health Organization (WHO), which proposes educational interventions targeting the public and healthcare professionals, such as public awareness campaigns [8]. Following the European Union (EU) Council Recommendation on the prudent use of antimicrobial agents in human medicine in 2001, stating that EU Member States should inform the general public of the importance of prudent use of antimicrobial agents, the European Centre for Disease Prevention and Control (ECDC) established the European Antibiotic Awareness Day (EAAD) in 2008. Various educational materials are available via the platform [9] to support national campaigns across countries [1,10].

After the country-specific recommendation released by the ECDC in order to tackle AMR and promote the prudent use of antimicrobials [11], in 2017 Italy adopted the first National Action Plan on Antimicrobial Resistance (Piano Nazionale per il Contrasto dell’Antimicrobico-Resistenza, PNCAR 2017–2020) [12]. The plan requires integrated and well-coordinated actions globally, nationally, and regionally, as well as at local government and institutional level, both in the human and in the veterinary fields, through a “One Health” strategy. The plan is a multicomponent tool that provides specific objectives and actions to be implemented at each level to promote effective strategies in different areas, including information and education of the general population through communication campaigns, as well as research and development [12].

The same year, the Sicilian Health Authority, Sicilian Region (Southern Italy), implemented a Regional Action Plan on prevention of HAIs, AMR, and inappropriate use of antimicrobials [13]. This plan includes a program for monitoring antibiotic consumption and resistance, as well as HAIs, in order to identify and define strategies and actions to fight antibiotic resistance [6]. Particularly, an education campaign, called “Obiettivo Antibiotico”, was promoted [14]. In this paper we present the planning and setup of the “Obiettivo Antibiotico” Campaign to raise awareness of prudent use of antibiotics in the public and healthcare professionals in Sicily.

## 2. Materials and Methods

### 2.1. Campaign Design

The educational campaign “Obiettivo Antibiotico” was launched in November 2018. The campaign aimed to increase awareness and promote engagement in order and to change behavior towards the rising threat of AMR in four main categories of target audience: (i) the general public (e.g., adults, parents, and students); (ii) primary care prescribers (i.e., general practitioners and pediatricians); (iii) pharmacists; and (iv) professionals in hospitals and other healthcare settings (i.e., nurses, microbiologists, clinicians, infection prevention and control staff, hospital pharmacists, and professionals working in emergency departments, intensive care units, and long-term care facilities, as well as managers/administrators).

The development of the campaign was led by the University of Catania, with the endorsement of the EAAD-ECDC [15], the Italian Ministry of Health, the Italian Society of Hygiene, Preventive Medicine and Public Health (SItI), the Sicilian Region, and the University Hospital "Policlinico-V. Emanuele”, Catania, Italy. 

The “Obiettivo Antibiotico” Campaign was designed by an interdisciplinary academic team of public health epidemiologists, sociologists, IT, and communication experts, integrating computer science and social sciences for public health: the so-called computational health science approach [16]. This approach allows taking advantage of the opportunities offered by social media and IT to address complex problems, such as AMR, and to inform public health practice directed at changing health behavior and the appropriate antimicrobial usage, through improved surveillance, communication, promotion, and targeted intervention. An interactive website [17], populated with different materials, was created and established online. The resources and toolkits of the campaign were selected from those developed by the EAAD [9], and included: key messages, letters, slogans, posters, factsheets, leaflets, and videos. Particularly, four EAAD toolkits were used and adapted: for primary care prescribers; for professionals in hospitals and other healthcare settings; for the general public; and, on self-medication, for the general public, pharmacists, and primary care prescribers. The key messages for each toolkit were derived from a thorough review of the scientific literature by ECDC experts. The toolkit materials were further updated and adapted to the regional epidemiological scenario with data from the regional “Surveillance of antibiotic consumption in the community and hospitals and surveillance of antimicrobial-resistance” [6], as well as with key findings on the levels of knowledge of the Italian population from the 2016 Eurobarometer survey [18].

The EAAD graphics were retained in order to maintain a common visual identity to the campaigns throughout Europe and to make the messages even more recognizable and consistent.

The website was the core intervention delivery tool, designed as a single scrolling page. The website’s homepage welcomes visitors with appropriately tailored videos, embedded at the top of the campaign website, to explain antibiotic importance in simple terms. The website was developed based on that of the UK “Antibiotic Guardian Campaign” [19], comprising an online pledge system in order to maximize the user’s engagement. Online pledges of the “Obiettivo Antibiotico” campaign aim to give every individual the opportunity to make concrete commitments to fight AMR. Pledges were designed to encourage behavior change around antibiotic prescription and antibiotic use. Through an interactive portion of the website, users could commit with a set of pledges (maximum of three) from a list tailored for each target audience.

This allows an in-depth analysis about which pledges are more relevant for the different profiles. Furthermore, the user can express custom pledges, and this also gives us more data analysis power to better refine messages for the different profiles. The idea behind the website design was to make the users feel as if they were an active part of the overall campaign.

For the design of the pledge portion of the website, various social digital interaction paradigms were considered in order to make sure visitors could find it intuitive and easy to navigate.

All data collected by the “Obiettivo Antibiotico” website were properly stored in a relational database structure. This allows us an easy way to slice and dice the collected data in order to support any data exploration. Furthermore, both the website and the underlying database structure were designed in order to allow the same system to be easily adapted to other contexts.

### 2.2. Social Media

Several criteria and metrics were considered to choose the most suitable social channels/platforms to use: how much of the population they can reach; the adaptability to different segments of the population; how reliable they can be in not generating consequences other than those desired; how much recipients can participate actively; the ability they have to influence the agenda-setting.

Various social media, including Facebook, Instagram, LinkedIn, and Twitter, were then selected. The campaign was an all-digital social campaign with well-thought-out material to drive traffic to the website. The key of the campaign was on “sensitize everyone” through the proper communication to experts (such as medical doctors and pharmacists) and the general public.

The planned and implemented communication and information activities included a time frame from 12 November to 21 December 2018. Ten posts per week were produced during this time.

### 2.3. Promotion

To encourage region-wide participation in the new campaign across Sicily, the “Obiettivo Antibiotico” Campaign was launched on 12 November 2018 to coincide with the EAAD, which is held annually on 18 November during World Antibiotic Awareness Week (WAAW), to join the initiatives that were taking place across Europe and globally during this period, to spread the messages on the risks associated with the inappropriate use of antibiotics.

The coordinator of the Campaign and the Department of Health of the Sicilian Region sent letters, by e-mail, to local health authorities, professional organizations, and scientific societies to engage stakeholders to support and disseminate the campaign. The campaign was promoted via social media and web-sites of involved institutions (the health authority and university). Several interviews were conducted with members of the interdisciplinary academic team. Five 2-min promotional videos were then produced from the interviews which were aimed at the public and hosted on the Facebook page and on a dedicated YouTube channel.

### 2.4. Data Collection

Visitors became part of the campaign by selecting up to three pledges on the “Obiettivo Antibiotico” website, providing personal data (name, province of residence, email address) and choosing an option for how they had heard about the campaign. Visitors were given the option to consent for future contact regarding campaign issues.

Google analytics tracking codes were input on 2 November 2018, three weeks after the website was online. The analytics were set to track all data with particular reference to the pledges made by visitors. Data were then analyzed for all website visits and acquisition routes via which visitors arrived to the website. Data were analyzed by descriptive statistics using Microsoft^®^ Excel 2018 (Microsoft Corporation, Washington, DC, USA).

## 3. Results

The main results achieved, from 12 November to 21 December 2018, through the campaign using the website and social media as communication tools, are summarized as follows.

### 3.1. Website

The campaign website had a total of 1159 unique visitors, of which 190 became champions of the campaign by choosing one or more pledges, with an overall conversion rate of 16.4%. While we consider this conversion rate quite satisfactory, we believe we can still improve the user experience to further improve conversion.

Visitors were mainly from Italy (91.57%). Google analytics showed that more than half of all website traffic was self-directed via social media (63.2%). The most visited pages were: “Home” with 1029 visitors, “Become one of us” (e.g., take a pledge) with 463 visitors, and “Hospital doctors” with 248 visitors.

**Figure 1:**
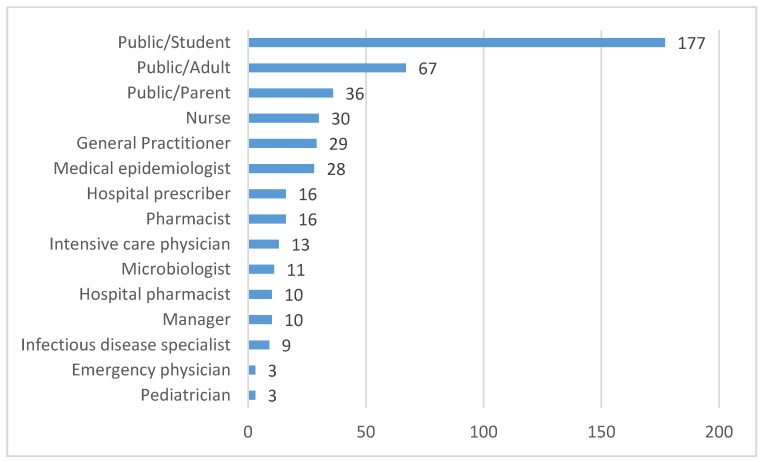
Number of pledges by target audience category/subcategory.

The total number of pledges made by the target audiences was 458, of which 61% were from the general public and 39% from experts. The breakdown of engagement by audience groups can be seen in Figure 1.

The highest participation was by far from the general public, with students choosing 177 pledges (39% of the total pledges taken). For this subcategory, the two most frequent selected pledges were the following:

1. “*The next time I see that a recommended practice for infection prevention is not respected (e.g., hand hygiene), I will point it out to my friends or colleagues and health professionals*”
2. “*For infections that my body can fight naturally, such as cough, cold, sore throat, and flu, I pledge to seek advice from my doctor/pharmacist about how to relieve symptoms.*”.

Professionals in hospitals and other healthcare settings, on the other hand, from among the different commitments, chose more frequently to spread the materials present on the website, trying to reach the greatest number of colleagues through the use of social media:

“*I will disseminate the materials of the campaign to raise awareness among my colleagues and patients, for example through social media and/or dissemination of information leaflets.*”

### 3.2. Social Media

Data from social media (Facebook, Instagram, Twitter, and LinkedIn) show that the audience is between the ages of 18 and 54 years; 64% female, 36% male; and mainly from Sicily. In terms of followers, LinkedIn achieved a particularly significant result with over 1200 connections, followed by Facebook with 675 followers. The lowest numbers were for Instagram and Twitter, with 82 and 16 followers, respectively.

**Figure 2:**
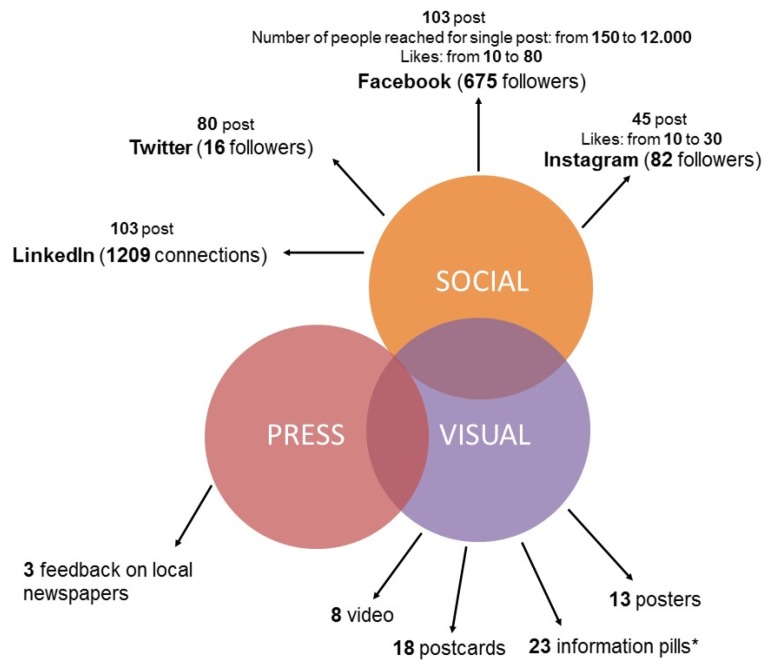
Types and distribution of media data. * The term “information pills” is a word play, referring to short key and easy-to-read (as simple as taking a pill) messages on the prudent use of antibiotics.

As for Facebook, the number of people reached by each post (i.e., a specific message on the social media to a specific group of friends) ranges from 150 to 12,000 (Figure 2), a result that we consider to be of interest.

## 4. Discussion

Surveillance data show that AMR is a rising public health problem in European hospitals and communities. The estimated burden of infections with antibiotic-resistant bacteria in Europe was reported to be similar to the cumulative burden of influenza, tuberculosis, and HIV. Italy has a substantially higher estimated burden than other European countries and about one-third of deaths due to infection with antibiotic-resistant bacteria in Europe occurred in Italy. Strategies to prevent and control AMR require coordination at the global level, and interventions that are tailored to national and local challenges [20]. In Italy, in 2017, the PNCAR was established in order to face the alarming AMR problem using the One Health approach [12]. Accordingly, in Sicily a regional surveillance system was implemented to monitor trends of HAIs, antibiotic consumption (both in the hospital sector and in the community), and antibiotic resistance [6,21]. Communication to the public on the association between unnecessary antimicrobial use and the emergence and spread of AMR seems an important component of strategies to control AMR [22]. In addition, addressing practices of healthcare professionals plays a fundamental role in tackling AMR. To ensure a prudent use of antimicrobials, the European Commission has conducted Eurobarometer surveys in order to monitor attitudes and behaviors of the public on the usage and knowledge of antibiotics [1]. Eurobarometer investigates the use of antibiotics among the general public, the levels of public knowledge about the nature and effectiveness of antimicrobials, and the risks associated with their unnecessary use, in Europe. The 2018 survey reported that the use of antibiotics varies by country, but it was observed that it is highest in Italy (47%), and lowest in Sweden (20%) and the Netherlands (21%). Most respondents obtained their last course of antibiotics from a healthcare professional (93%) and around 7% of antibiotics were taken without a prescription. Bronchitis, sore throat, flu, and urinary tract infection are the most common reasons mentioned for taking antibiotics. A total of 85% of survey respondents are aware that unnecessary use of antibiotics makes them become ineffective, but less than half (43%) of respondents know that antibiotics are ineffective against viruses [1].

Within this framework, in 2018 the “Obiettivo Antibiotico” Campaign was designed and launched to raise awareness of the prudent use of antibiotics in the public and healthcare professionals in Sicily [14]. Preliminary results of this experience show, for the first time in Italy, measurable engagement in a public health campaign. The literature suggests that commitment to a pledge is significant towards motivating behaviour change. This might be enhanced when people share their commitment openly and further when the individual feels part of a community [23]. The role of the Internet and social media as a promoter of health issues was confirmed by previous research showing that websites on health can play a significant role in raising public awareness [24,25,26]. Interestingly, as previously demonstrated in other contexts, often citizens’ concern is not a function of a real risk, thus it is necessary that the media themselves cooperate with public health authorities to keep risk perception at the real level of the hazard [27].

One of the main results of our work is that the highest participation was by far from the general public, especially from students, and on the contrary less involvement was observed for healthcare professionals. Engagement through social media provides an opportunity to exchange on health matters and real-time learning [28]. The use of social media in students’ and young adults’ everyday lives is now ubiquitous, and research in the field of education emphasizes that more use of such technologies in academic contexts would lead to increased engagement [29,30,31]. A significant implication that emerges from this study is that the high level of engagement of students represents an important achievement of the campaign, as they will have the opportunity to influence the future healthcare workforce. Thus, in the future an appropriate approach should be developed further to engage with other groups, including those without prior knowledge of AMR. Finally, to further encourage participation amongst healthcare professionals across Sicily, and achieve a more active commitment with primary and secondary care hospitals, engagement with directors of public health, local authority boards, and professional organizations should be planned through a coordinated regional promotional network.

Our results highlight the need for continued social media promotion in supporting behavioral change. It has been reported that in the Antibiotic Guardian campaign, healthcare professionals most commonly found out about the UK campaign through professional networks and channels, while the public was engaged most frequently via social channels [32]. This underlines the importance of maximizing the use of professional networks and the involvement of professional organizations and scientific societies to reach more members in the field of healthcare.

Our study has some limitations. First, we analyzed indicators of engagement for the first weeks after the launch of the campaign. However, the “Obiettivo Antibiotico” campaign was designed as an “always on” campaign, similar to the UK “Antibiotic Guardian Campaign”. Although, the launch and the promotional phase took place in November 2018, the website remains active and educational materials and information can be accessed anytime. Second, although it was a low-cost campaign, reliant on local and individual engagement and promotion activities, we did not evaluate the cost-effectiveness of the campaign. It should be noted that evaluation of the effectiveness of multicomponent interventions, such as information campaigns containing several interacting components, is difficult, and their generalizability of such interventions is problematic. Several factors could influence their success, in particular local context and strategy mix [33].

## 5. Conclusions

The present campaign represents the first attempt for a regionwide multicomponent campaign in Sicily, using social media. This study also fills a gap in the literature on how to develop an educational campaign and plan for assessment of the effectiveness of the campaign. Further in-depth analysis of the effect of the “Obiettivo Antibiotico” campaign on behavior change and, ultimately, antibiotic use with those who made a pledge, is needed. A follow-up evaluation is underway with people who pledged to become “one of us”. The ongoing study is a summative evaluation since it aims to detect some dimensions of effectiveness that will allow us to assess if and how the opinions and behaviors of the people involved have changed thanks to the information shared and the pledges made by the participants. To this end, a structured survey has been carried out and distributed among all individuals involved in the campaign. Data analysis is still ongoing at the time of writing of this paper. The results will also be used to further inform future changes to the “Obiettivo Antibiotico” campaign. However, evaluation of the campaign in terms of understanding its impact on antibiotic consumption and on AMR is difficult and its effects are unlikely to be immediate, as shown from previous national campaigns in some European countries [15]. Moreover, the impact of the campaign on reduced antibiotic use is not part of the present study. To extend the “Obiettivo Antibiotico” will require sustained effort, funding, and outreach activities. Furthermore, additional efforts are needed to reach more people. Thus, future efforts should focus on further promotion within the Sicilian region to sustain the engagement with the campaign.
